# Online symptoms self-assessment during COVID-19 pandemic: an analysis of a COVID-19 portal responses from Canada

**DOI:** 10.1038/s41598-022-13053-z

**Published:** 2022-05-31

**Authors:** Bonaventure A. Egbujie, Krizia Francisco, Mohamed Alarakhia, John P. Hirdes

**Affiliations:** 1 School of Public Health and Health Systems, University of Waterloo, Waterloo, Canada; 2 Ontario Health (West Region), Toronto, Canada; 3 eHealth Center of Excellence, Waterloo, Canada; 4 Michael G. DeGroote School of Medicine, McMaster University, Hamilton, Canada

**Keywords:** Health policy, Health services, Public health

## Abstract

COVID-19 case was first identified in Canada on January 25, 2020, on a Toronto resident who had travelled to Wuhan China, and not long after, the WHO declared the viral infection a pandemic. Ontario health West created an online self-assessment portal that allowed individuals in the health region and adjourning areas to report any COVID related symptoms. The purpose of this study was to evaluate the utility and usefulness of the Ontario Heath West online COVID-19 self-assessment portal. Record level data obtained from the Ontario Health West self-assessment portal was analyzed. Descriptive statistics using charts and graphs were used to characterize the distribution of responses to the portal. In-depth analysis using correlation, lead-lag analysis, and trend comparison with actual Government of Ontario COVID-19 cases for the region were also conducted. A total of 34,144 distinct responses were recorded on the portal between April 10 and July 29, 2020, with 1,250 (3.7%) responding positively to one of the emergency symptoms questions. Trend analysis showed a peak portal response in May 2020 with a smaller rise subsequently in July 2020, coinciding with the actual COVID-19 peak in the region. The five most reported symptoms on the portal were sore throat (17.2%), headache (12.9%), fatigue (12.3%), digestive problems (12.2%) and cough (9.1%). For four sub-regions, the trend of self-report on the portal positively lagged actual Public Health Ontario reported COVID-19 cases, while for one sub-region, the trend positively led the actual Public Health Ontario reported COVID-19 cases for the area. We found correlation between online COVID-19 self- assessment data and the confirmed COVID-19 cases in the Southwestern region of Ontario. Trends in the COVID-19 associated emergency symptoms reported on the portal also tracked confirmed COVID-19 cases in the community. Peak response to the portal coincided with the peak volume of confirmed cases in Ontario during the first wave of COVID-19 pandemic in Canada, suggesting some consistency between the experiences of portal users and patterns of COVID-19 illness in the community. The portal was a useful tool at the person-level because it provided guidance to individuals about how to access appropriate health services according to the symptoms that they reported and connected them with primary care, reducing unnecessary visit to health facilities for COVID-19 related care.

## Introduction

The first case of COVID-19 in Canada was identified on January 25, 2020, and involved a Toronto resident who had travelled to Wuhan, China^1^. The World Health Organization declared a global pandemic of Coronavirus disease (COVID-19) on March 11, 2020^2^. By March 24, 2020, Health Canada reported that local transmission was the primary cause for new cases in Canada with 2,792 cases confirmed by that point. There was a clear need for a systematic approach to managing the response to COVID-19 that would: (a) identify new cases in the community; (b) educate and guide the general population to help them to detect and respond appropriately to potential COVID-19 symptoms, and (c) provide a linkage mechanism that would provide a targeted referral for testing and clinical follow-up with their primary care based on those potential symptoms.

One aspect of the response undertaken by multiple jurisdictions in the world was to create online tools that would allow members of the public to self-assess their own health status to receive advice on what response may be appropriate given their results^3–5^. The Ontario Health West Region created such a tool in partnership with Input Health, and the system became fully operational in early April 2020. The portal which is now deactivated [URL link: http://lmcovid19.inputhealth.com] provided individuals in the region and other neighbouring regions with the opportunity to self-assess for COVID-19 related symptoms.

## Methods

We analyzed record level data obtained from the Ontario Health West Region self-assessment portal. All data were anonymous and do not include demographic measures (e.g., age, gender, race). Individuals who responded positively to any of the initial emergency symptoms screening questions did not receive further self-assessment screening on the portal and were therefore excluded from the comprehensive analysis. The initial emergency symptoms include a) severe difficulty breathing (Struggling for each breath, can only speak in single words), b) severe chest pain (Constant tightness or crushing sensation), c) feeling confused (for example, unsure of where you are) and d) losing consciousness with a No/Yes response. However, we performed a specific trend comparison between reported emergency symptoms and actual Public Health Ontario general population COVID-19 cases for the region. Information regarding the government of Ontario confirmed COVID-19 cases can be obtained by clicking on the link: [https://data.ontario.ca/dataset/confirmed-positive-cases-of-covid-19-in-ontario/resource/455fd63b-603d-4608-8216-7d8647f43350]. The symptoms variables were treated as binary categorical, and a K-Mode clustering algorithm was applied to find hidden clusters in the dataset.

Descriptive statistics including frequencies and bar charts were used to describe the comprehensive data. Spearman’s correlation was used to determine associations between the reported portal symptoms, while a plus-minus 3 weeks lead-lag correlation matrix was created to determine correlations between the number of people who reported symptoms on the portal and the number of actual reported COVID-19 cases per region. All analyses were restricted to responses from persons residing in the forward sortation area (FSA) beginning with ‘N’ representing southwest Ontario.

### Statement on guidelines

This study conforms to applicable and relevant guidelines. Being a completely anonymized, non-intervention online survey, no ethics approval was required. No identifying information was collected for this study and all analyses were conducted at the population level. This study was carried out using only data from the Ontario Health West self-assessment portal and there was no direct involvement of participants.

## Findings

### Response volumes

**Figure 1:**
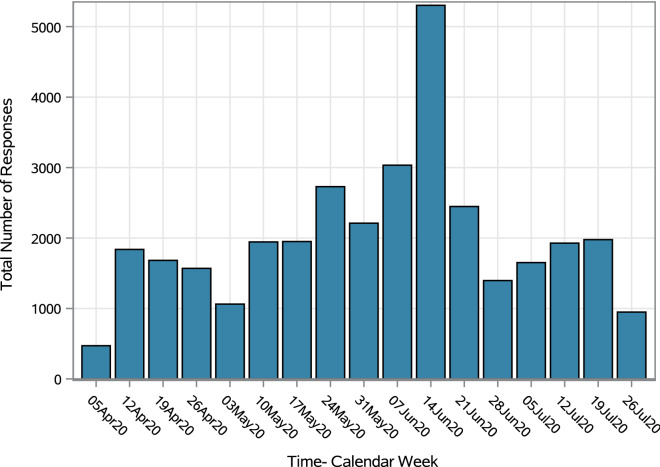
Number of responses to the COVID-19 Self-assessment portal over time, Ontario Health West.

A total of 34,144 distinct responses were collected between April 10 and July 29, 2020. Out of these, 1250 (3.7%) responses were positive for having any emergency symptoms and were subsequently referred to the next step in care management. A further 14,340 responses (42.0%) indicated the presence of other, non-emergency symptoms potentially related to COVID-19. Another 3112 (9.1%) responses indicated the presence of high-risk health conditions (e.g., heart failure, diabetes, age >  = 70, auto-immune disease, immunotherapy) without potential COVID-19 symptoms being present. Figure 1 shows the number of responses to the self-assessment portal by week. The distribution suggests that the peak inquiries on the platform occurred between May 24 and June 28, 2020, with the highest volume occurring in the 3^rd^ week of June.

### COVID-19 indicator patterns over time

**Figure 2:**
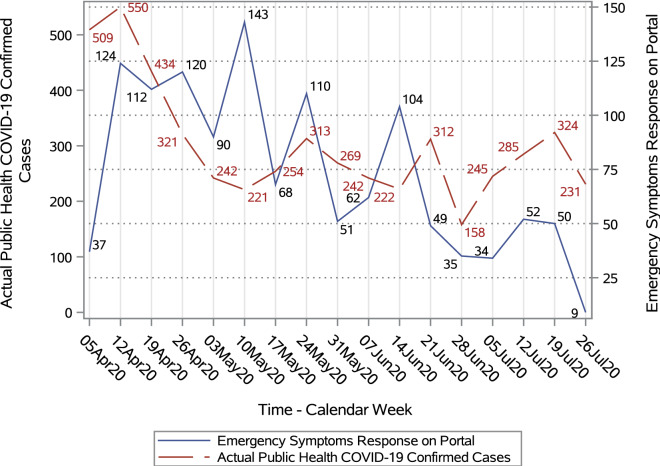
Trend in number of emergency COVID-19 symptoms on portal versus actual diagnosed COVID-19 cases.

**Figure 3:**
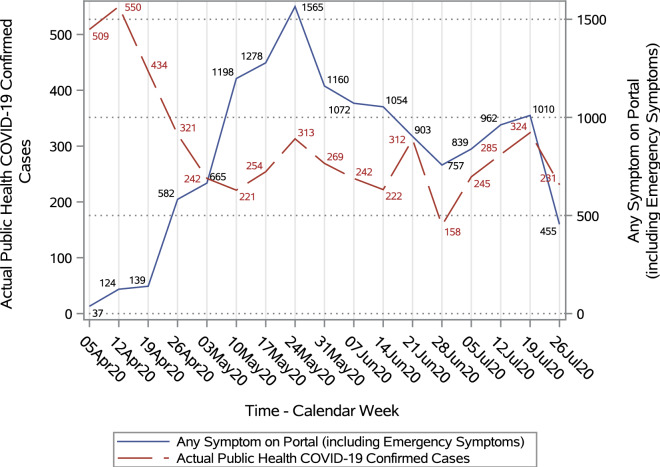
Trend in number of respondents with COVID-19 symptoms versus actual diagnosed COVID-19 cases.

Responses to the portal questionnaire differentiated between the presence of any of a set of emergency symptoms (e.g., shortness of breath) that would require immediate medical attention and other symptoms that may be indicative of COVID-19 but were not considered to require emergency care. Figure 2 shows the numbers of portal responses with emergency symptoms present as well as the number of actual COVID-19 cases reported by government of Ontario in the postal code (FSA) ‘N’ over same time. Figure 3 shows the corresponding distributions of any potential COVID-19 symptoms (emergency or other) and confirmed cases in the region. In both plots, two y-axis scales are shown to allow comparisons between the trends in portal responses and COVID-19 cases confirmed by governement of Ontario.

### Geographic response patterns

**Figure 4:**
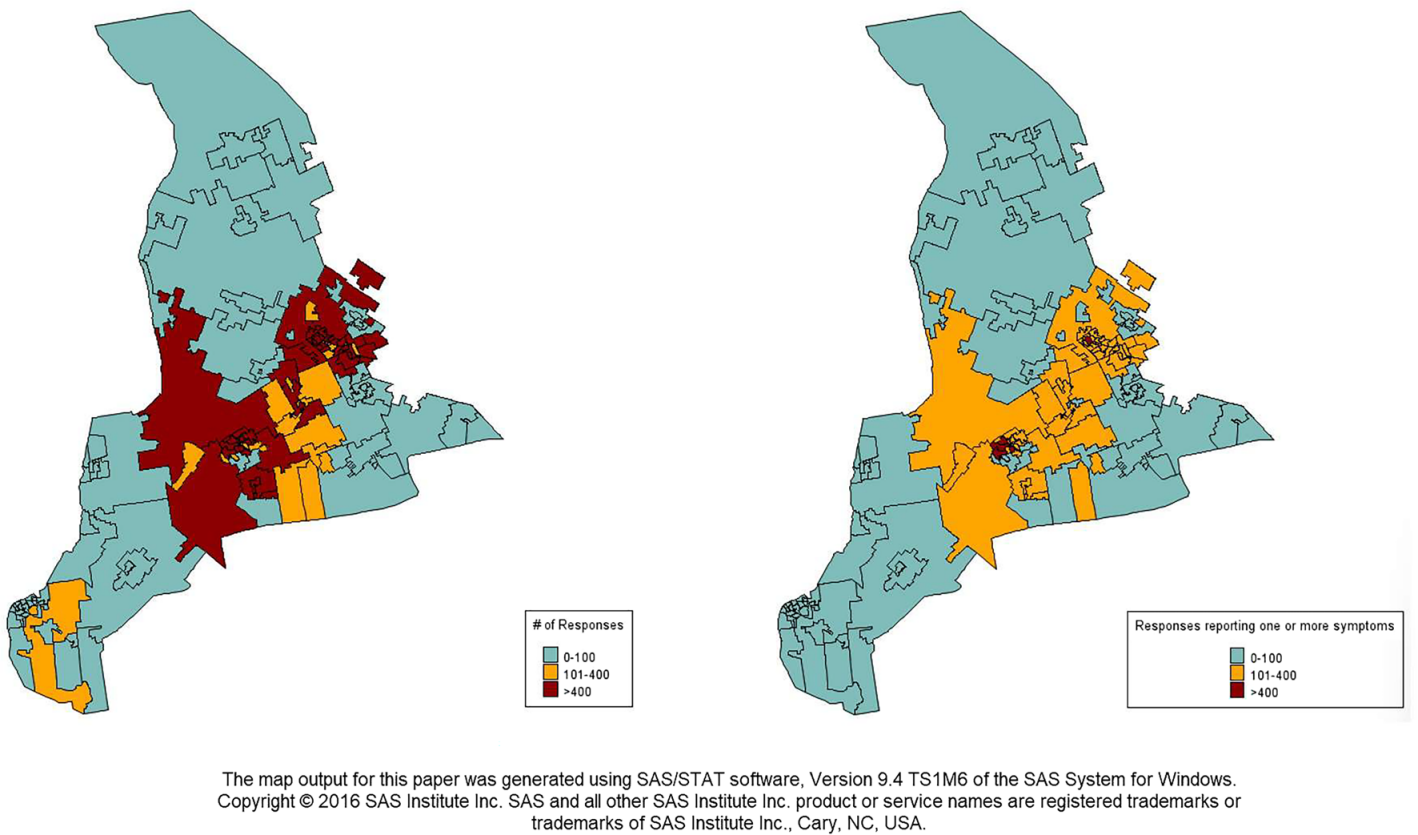
Geo-spatial distribution of portal responses and presence of at least COVID-19 related symptoms by F.

**Figure 5:**
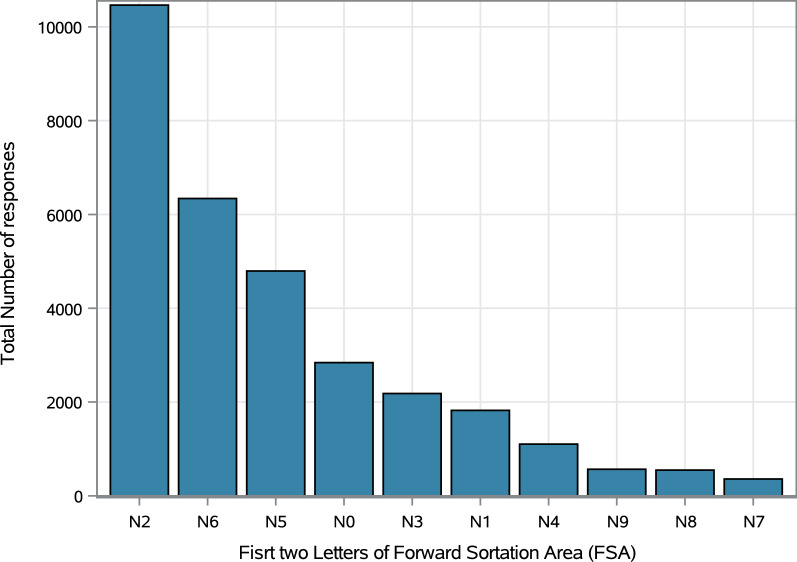
Number of portal responses by sub-regions from the ‘N’ postal code area.

**Figure 6:**
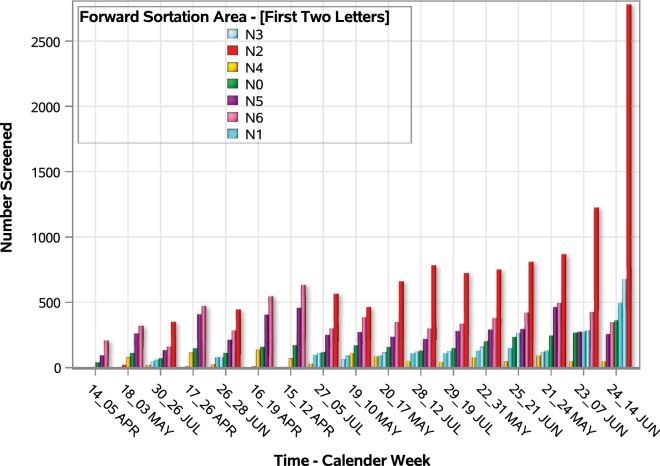
Trend in the number of self-assessments on the portal by postal code.

A total of 31,016 (99.6%) responses came from individuals who stated that they live in the FSA ‘N’ area, the primary catchment area for which the portal was set up. Figure 4 shows the geographical distribution of response volume, and the symptoms count distribution by FSA area. Because most responses came from the ‘N’ region, we limited our analysis to focus on this region. Within the ‘N’ FSA, the N2 region (see Online Appendix S1 for table of locations for each sub-region) recorded the highest absolute number of responses with 10,464 responses (Fig. 5), while the ‘N7’ region had the lowest absolute number with 358 responses during the same period. During the first 5 weeks of the data collection, the absolute number of responses from the ‘N6’ was the highest, but from the 6th week, the ‘N2’ region recorded the highest numbers per week. In the 11th week, the ‘N2’ FSA recorded an exponential increase in the number of responses (Fig. 6). This may be due to data error or a real surge in responses from this region in response to differential COVID-19 outbreak patterns, which will require further investigation to ascertain the origin.

**Figure 7:**
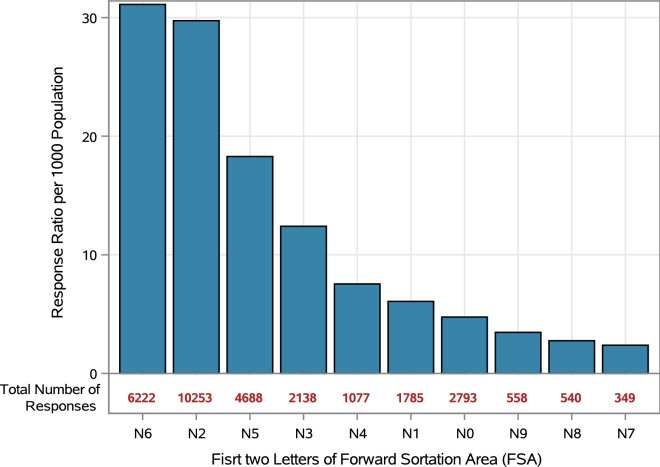
Portal responses per 1000 population by postal code sub-region.

To compute the comparative response levels between the FSAs, we used Statistics Canada’s 2016 census data^6^ to estimate the number of responses per 1000 residents in each postal code sub-region. FSA beginning with N6 had the highest per capita response, whereas those beginning with N7 had both the lowest per capita response (Fig. 7).

### Symptoms reported by portal respondents

**Figure 8:**
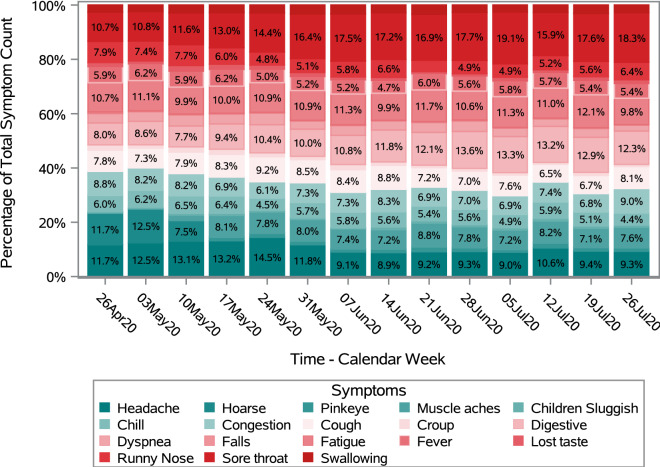
Percentage of respondents reporting COVID-19 related symptoms in FSA ‘N’ area.

**Figure 9:**
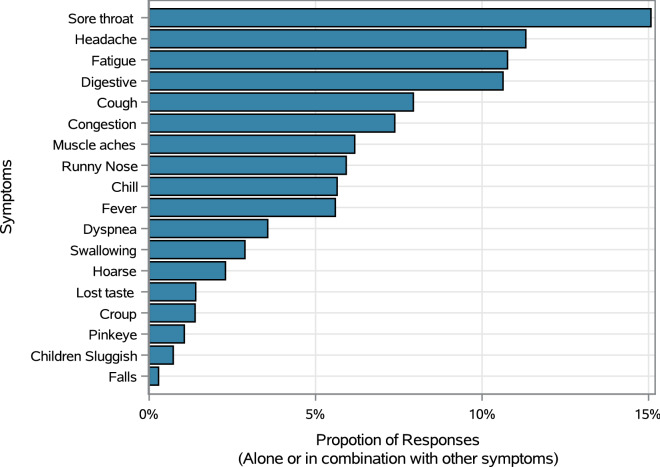
Frequency of COVID-19 related symptoms reported on the portal.

Figure 8 shows the trends in symptoms reported over time by type of symptom. All symptoms peaked in early May with a decline thereafter; however, a smaller, brief secondary peak was evident at the end of July. Sore throat was the most common symptom reported by respondents with 5880 of 31,016 non-emergency symptom respondents reporting they have the symptom either alone or in combination with other symptoms. The five most reported symptoms (Fig. 9) were sore throat (17.2%), headache (12.9%), fatigue (12.3%), digestive problems (12.2%) and cough (9.1%).

**Figure 10:**
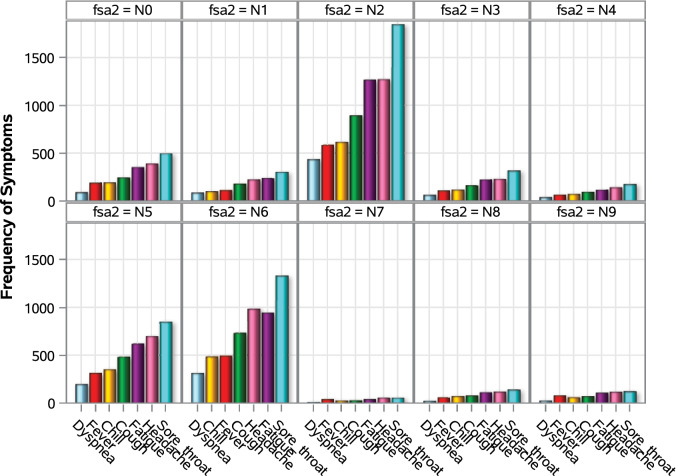
Distribution of the most common symptom types reportd on the portal by postal code.

In terms of volume of the symptoms, respondents from FSA N2, N5 and N6 reported the most symptoms, while respondents from FSA N8 and N9 reported the most average symptom per respondents. However, further analysis showed that the distribution of symptoms within each FSA was largely similar (Fig. 10).

### Symptom counts and clusters

There were regional variations in counts of symptoms reported in the Ontario Health West COVID-19 portal. The most common pattern was that respondents had no symptoms recorded; however, this is an artefact of two issues. First, if the respondent had any *emergency* symptoms, they were not asked any further questions about other symptoms or their health region. Instead, they were advised to seek immediate medical attention. Second, persons who had travelled to regions affected by COVID-19 or who had been in contact with persons with COVID-19 were not asked symptom-related questions. Both subgroups are present in the zero symptoms group.

On the other hand, up to 30 percent of respondents in some subregions reported three or more potential COVID-19 symptoms. There are regional variations evident in the symptom counts, but caution should be exercised in the interpretations of these differences because of the challenge in differentiating respondents who truly had no symptoms from those who were not asked the full set of questions about symptoms due to skip logic built into the portal.

Using Spearman rank sum correlation matrix, we examined patterns of associations between reported symptoms. The results suggest that fatigue tended to be accompanied by reports of chills, headaches, digestive symptoms, and sluggishness among children. Not surprisingly, fever and chills were associated with each other, as were congestion and runny nose. Following the implementation of a K-Mode machine learning clustering algorithm to the dataset to identify additional clusters, no other clusters were found beyond those evident from the correlational analyses in Table 1. It was not possible to conduct classification (supervised) machine learning modelling with the data. In part, machine learning performance was further limited by the lack of outcome or criterion measures (labels) that could be linked to symptoms at the person level.

**Table 1:** Lead-Lag correlation matrix symptoms reported on the portal vs actual COVID-19 cases (per 1000 population) per sub-region.

	Guelph Cambridge Dunnville	Kitchener-Waterloo	Cambridge Brantford Elmira Paris	Woodstock Stratford Owen Sound Hanover Delhi	Stratford London St. Thomas Ingersoll	London	Sarnia Chatham-Kent Goderich	Windsor La Salle
	N1	N2	N3	N4	N5	N6	N7	N9
Headache	No positive correlation	No positive correlation	1 week lead			3 week lag	2–3 week lag	No positive correlation
Cough						3 week lag		
Runny nose			1 week lead		3 week lag	3 week lag		
Dyspnea			2 week lead	3 week lag		3 week lag	3 week lag	
Swallowing difficulty							3 week lag	
Digestive problem								
Sore Throat								
Chills						2–3 week lag		
Lost taste			2 week lead*			3 week lag		
Fatigue					3 week lag	2–3 week lag		
Fever								
Number assessing portal						1–2 week lag		

### Linkages to primary care

An important function of the COVID-19 self-assessment portal was to connect persons with potential symptoms to their primary health care provider when appropriate. Among 15,619 responses with any symptoms present (excluding emergency symptoms), 74.2% (11,589) had a primary health care provider that they were referred to for follow-up. These respondents were able to avoid emergency room visits and directed to contact the COVID-19 assessment center, because they could be appropriately served by primary care based on their response profile. About 17% of responses with symptoms had no primary health care provider; however, they were supported through access to an on-call provider.

### Relationship between portal symptom reporting and sub-regional outbreaks

Following the initial analysis suggesting similar trends between number of respondents with symptoms on the portal self-assessment and actual number of COVID-19 cases in the region, we conducted further in-depth statistical analysis of this association. We standardized the number of portal symptoms and COVID-19 case by computing each number per 1000 population of the respective postal code. To ensure we covered for possible temporal variations in association, we computed up to 3 weeks lag and lead correlations between the portal symptoms and actual COVID-19 outbreaks.

For all the sub-regions, the number of people who reported each symptom were mostly *negatively* correlated with the number of COVID-19 cases. However, in a few sub-regions there were some significant positive correlations between number of symptoms reported through the portal and the actual number of COVID-19 cases reported to government of Ontario.

In regions with first two digits of the postal code, ‘N4’–‘N6’, the symptoms inconsistently showed significant positive correlation with actual number of COVID-19 cases for the region, with a 2–3 weeks lag time. This seems to suggest that in these sub-regions, portal symptoms reports started to rise about 2 or 3 weeks *after* the increase in actual COVID-19 cases reported to government of Ontario for each of the sub-regions. This suggests that individuals came to the portal in response to outbreaks happening in their sub-region.

Conversely, for sub-regions with the first two digits of postal code of ‘N3’, there were some positive correlations between some symptoms and actual COVID-19 cases, but with a lead time suggesting that people increasingly began to report such symptoms on the portal one to two weeks *before* COVID-19 cases began to rise in the area. Table 1 shows symptoms that were found to have positive lead or lag correlations with COVID-19 cases per sub-region. Some sub-regions, however, did not have any positive association with actual COVID-19 cases.

## Discussion

In this analysis, we found some correlation between online COVID-19 self- assessment data and an actual confirmed COVID-19 cases in the Southwestern region of Ontario. Although trends in the two self-assessments (emergency symptoms and any symptoms) and actual COVID-19 indicators were not identical, there was a somewhat consistent pattern between the portal symptom responses and confirmed cases in the community. Peak response to the portal coincided with the peak volume of confirmed cases in Ontario during the first wave of COVID-19 pandemic in Canada^7^. This does not suggest the portal data could be used for epidemiological purposes, but it does suggest some consistency between the experiences of portal users and patterns of COVID-19 illness in the community.

The inconsistent picture obtained from the analysis of portal responses may have been due to several reasons. First, awareness and utilization of the portal among the general population in the region of may have been low. Reduced utilization rates compared to the population size could potentially bias the finding towards the characteristics of only those who are aware of the portal.

Secondly, the different correlations showing lead in some regions and lag in others, might reflect the actual difference in epidemiology and management response to COVID-19 by region during the period under this review. Different regions (though more regional and national) began to experience COVID-19 cases at different times and the rate and speed of detection also differed by the sub-regions. The picture obtained through the Ontario Health West Region portal may suggest that postal code areas in the region experienced their COVID-19 outbreaks differently. Also, testing and detection may have proceeded at different pace and coverage during the 1st wave of the pandemic. Therefore, these results suggest that caution should be used with respect to the utility of such a portal in foretelling subsequent infection patterns in the population.

While the Ontario Health West COVID-19 self-assessment portal had some limitations that constrained its utility for public health or epidemiological reporting, it served a valuable health system function in providing the public with an accessible source of information about COVID-19 and linked them to appropriate services they may use like a primary care assessment. The patterns evident in our early analyses suggested that there was some tendency for response patterns in the portal to mirror trends related to COVID-19 reported by government of Ontario. However, there was an inconsistent relationship between symptom reporting on the portal and subsequent COVID-19 outbreaks. Nonetheless, at the person level, this portal served a useful health system function in helping individuals to better understand their health needs and the services they may require.

With respect to any future waves of the COVID-19 pandemic, we would note the following recommendations for improvement of this or similar portals. First, these portals should capture information on demographic characteristics including age, sex, and ethnicity, since it is known that COVID-19 affects different subgroups with differing severity and different event rates for adverse outcomes. Second, skip patterns should generally be avoided. The small-time savings gained by not gathering additional information is not worth the loss of information that could have informed symptom profiling or severity measures. Third, it is essential to track clinical and service use outcomes. The portal did not allow adequate linkages to determine what levels of service use or clinical outcomes were associated with the symptom characteristics reported in the portal. This limited the ability to use statistical analyses or machine learning to identify important subgroups of respondents that may have warranted additional attention based on symptom clusters. Fourth, while the portal had limited population level applications for epidemiological tracking or analyses, it was a useful tool at the person-level because it provided guidance to individuals about how to access appropriate health services according to the symptoms that they reported and connected them with primary care. In that sense, continued public access to portals of this type for subsequent waves of COVID-19 could be beneficial.

## Supplementary Information

Supplementary Information.

## Data Availability

The datasets used and/or analyzed during the current study are available from the corresponding author on reasonable request.
