# Supplementary material for: Online symptoms self-assessment during COVID-19 pandemic: an analysis of a COVID-19 portal responses from Canada
Source: Sci Rep. 2022 May 31;12:9036. doi: 10.1038/s41598-022-13053-z (PMC9156693; doi:10.1038/s41598-022-13053-z)
Supplement: Supplementary file 1 — Supplementary Information. [file 41598_2022_13053_MOESM1_ESM.pdf]

Appendix 1. Postal codes and coverage areas used for analysis

N0	N1	N2	N3	N4	N5	N6	N7	N8	N9
RURAL AREAS	Dunnville	Kitchener East	New hamburg	Delhi	Stratford North	London North (UWO)	Goderich	Wallaceburg	Windsor (City Centre / NW Walkerville)
	Guelph South	Kitchener Northeast	Elmira	Tillsonburg	Ingersoll	London Central	Strathroy-Caradoc	Leamington	Windsor (University / South Cameron)
	Guelph North	Kitchener South Central	Cambridge Northeast	Owen Sound	Aylmer	London South (East Highland / North White Oaks / North Westminster)	Chatham-Kent Northwest	Essex	Windsor (Sandwich / Ojibway / West Malden)
	Guelp Central	Kitchener West	Cam ridge Northwest	Meaford	Port Stanley	London (South White Oaks / Central Westminster / East Longwood	Chatham-Kent Southeast	Tecumseh Outskirts (Windsor)	Windsor South (East Malden)
	Guelph Northwest	Kitchener Central	Cambridge West	Hanover	St.Thomas North	London (Sunningdale / West Masonville / Medway / NE Hyde Park / East Fox	Samia Central	Windsor (East Riverside)	Windsor (Roseland)
	Guelph West	Kitchener North Central	Paris	Woodstock Central	St. Thomas South	London West (Central Hyde Park / Oakridge)	Samia Southwest	Windsor (East Forest Glade)	LaSalle East (Windsor)
	Fergus	Kitchener Southeast	Brantford Northeast	Woodstock North	London (YXU / North and East Argyle / East Huron Heights)	London (Southcrest / East Westmount West Highland)	Samia Northwest	Windsor (Riverside)	La Salle West (Windsor)
	Cambridge Central	Waterloo East	Brantford Central	Woodstock South	London East (SW Argyle / Hamilton Road)	London (Riverbend / Woodhull / North Sharon Creek / Byron / West Westmount	Samia Southeast	Windsor (West Forest Glade / East Fontainbleu)	Windsor
	Cambridge Southwest	Waterloo South	Brantford Southeast	Litowel	London (Fanshawe / Stoneybrook / Stoney Creek / Uplands / East Masonville)	London (East Tempo)	Samia Northeast	Windsor (YQG)	Amherstburg
	Cambrideg East	Kitchener Northwest	Brantford Southwest	St. Marys	London (West Huron Heights / Carling)	London (Jackson / Old Victoria / Bradley / North Highbury)		Windsor (South Walkerville / West Fontainbleu / Walker Farm / Devonshire)	Kingsville
		Kitchener South	Brantford Northwest	Stratford South	London (Glen Cairn)	London (South Highbury / Glanworth / East Brockley / SE Westminster)		Windsor South Central (West Walkerville / Remington Park)	
		Waterloo Southwest	Caledonia			P London (Talbot / Lambeth / West Tempo / South Sharon Creek)		Windsor East (East Walkerville)	
		Waterloo Northwest	Simcoe						
